# Decreased Management of Genital Warts in Young Women in Australian General Practice Post Introduction of National HPV Vaccination Program: Results from a Nationally Representative Cross-Sectional General Practice Study

**DOI:** 10.1371/journal.pone.0105967

**Published:** 2014-09-02

**Authors:** Christopher Harrison, Helena Britt, Suzanne Garland, Lynne Conway, Alicia Stein, Marie Pirotta, Christopher Fairley

**Affiliations:** 1 Family Medicine Research Centre, School of Public Health, University of Sydney, Sydney, Australia; 2 Microbiology & Infectious Diseases, The Royal Women's Hospital, Parkville, Australia; 3 Health Economics, bioCSL, Parkville, Victoria, Australia; 4 bioCSL, Parkville, Victoria, Australia; 5 Department of General Practice, University of Melbourne, Parkville, Victoria, Australia; 6 Central Clinical School Monash University and Melbourne Sexual Health Centre, Carlton, Victoria, Australia; Albert Einstein College of Medicine, United States of America

## Abstract

**Objectives:**

Since the introduction of Australia's human papillomavirus vaccination program, the management rate of genital warts in sexual health clinics and private hospitals has decreased in women of vaccine-eligible age. However, most genital warts in Australia are managed in general practice. This study examines whether a similar decrease occurred in Australian general practice after the introduction of the program.

**Methods:**

Analysis of a nationally representative cross-sectional database of Australian general practice activity (1,175,879 patient encounters with 11,780 general practitioners). Genital warts management rates were estimated for the periods before and after introduction of the program (Pre-program, July 2002-June 2006; Post-program, July 2008-June 2012). Control conditions included genital herpes and gardnerella/bacterial vaginosis in female patients and genital herpes and urethritis in male patients. Trends in management rates by year, pre-vaccine (July 2000-June 2007) and post-vaccine (July 2007-June 2012) were also calculated.

**Results:**

Management rate of genital warts among women potentially covered by program (aged 15–27 years) decreased by 61% from 4.33 per 1,000 encounters in the Pre-program period to 1.67 in the Post-program period. Trend analysis of the post-vaccine period showed, among women of vaccine eligible age, a significant year-on-year reduction in the rate of genital warts management (p<0.0001) and a significant increase in the management rate of control conditions per year (p<0.0001). For all other age-sex groups there was no significant change in the management rate of genital warts between the Pre- and Post-program periods.

**Conclusion:**

The large decrease in general practice management of genital warts in women of vaccine-eligible age highlights the success of the program in the wider community.

## Introduction

Australia was one of the first countries to provide the human papillomavirus (HPV) vaccine free to young women through a national immunisation program. The program was initially promoted in terms of prevention of cervical cancer, as HPV types 16 and 18 are known to be the major aetiological agents to cervical cancer in around 70% of cases worldwide.[1], [2] However, the quadrivalent vaccine also protects against HPV types 6 and 11, the major causes of genital warts.[3]–[5] Genital warts cause distress in those patients affected by them[6] and have a substantial treatment cost.[7]

In April 2007, the Australian Government introduced an ongoing free school-based program using the quadrivalent HPV vaccine (Types 6, 11, 16, 18) for girls aged 12–13 years and a two year catch-up program for girls aged 13–18 years in a two year catch-up.[8] In July 2007, a community-based catch-up program for women aged 18–26 years (inclusive) was initiated, with the HPV vaccine available free through general practitioners (GPs) and other primary health care services until December 2009.[9] The program has been very successful with the National HPV 3-dose vaccination coverage for all females turning 15 years of age ranging from 72.5% in 2007 to 70.9% in 2012. Similar coverage rates (between 70% and 72%) have been shown for females aged 16 to 17 years. Coverage was lower for the older populations, although it has been progressively increasing, from 39% in 2007 to 69% in 2012 for females aged 18 to 19 years and from 30% to 44% in females aged 20 to 26 years over the same period.[10]

The quadrivalent HPV vaccine was also approved in Australia for older women (aged 27–45 years) and for males aged 9–26 years. However it was not subsidised for these groups and coverage has been estimated as very low; the coverage rate for senior high school boys (aged 15–17 years) was <5% in 2008 compared with >85% in girls of the same age.[11] In February 2013, the Australian Government extended the free ongoing school-based quadrivalent HPV vaccination program to include boys aged 12–13 years, with a two year catch-up program for boys aged 14–15 years.[12]

Donovan al found that with the introduction of the HPV vaccination program in 2007, in sexual health clinics the rate of genital wart diagnosis decreased significantly and quickly among women who would have been covered by the program (those aged 12–26 years in 2007). There was also a slight, but significant, decrease in the incidence of diagnosed genital warts among heterosexual men, especially those in the same age cohort as women covered by the program. This decrease was attributed to herd immunity from the female vaccination program. The study showed no effect on the rate of genital wart diagnosis in men who had sex with men (MSM).[13] In a recent update of these clinics, it was reported that for vaccine-eligible age girls, diagnosis of genital warts had decreased by 93% in those aged less than 21 years and 73% for those aged 21–30 years. For males in these age groups, the decreases were 82% and 51% respectively.[14] There was also significant decrease in the management of genital warts in women aged 15–34 years and to a lesser extent men of the same age in private hospitals.[15]

However, only 17% of genital warts cases are managed in sexual health clinics and only 7% are managed in a hospital setting, with the majority (57%) being managed in general practice.[16] Australia has a universal medical insurance scheme called Medicare which (fully or partially) covers the individual's cost of visits to GPs. Additionally, GPs provide the bulk of primary medical care and act as gate keepers to government-subsidised health care from other medical specialists. There are no patient lists and patients are free to visit multiple GPs and practices as they choose. The aim of this paper is to examine whether the decrease in genital warts incidence and management found in patients attending sexual health clinics and private hospitals also occurred in the broader population of patients attending general practice.

## Methods

To evaluate changes in the management rate of genital warts in general practice we used data from the Bettering Evaluation and Care of Health (BEACH) program.[17] The BEACH program began in April 1998 and is a continuous cross-sectional, national study, collecting details of the content of GP-patient encounters in Australia. Every year, each of approximately 1,000 randomly selected general practitioners (GPs), records details of 100 consecutive encounters on structured paper recording forms, including problems managed, tests ordered, and treatments provided. Also BEACH provides information about the patient, the GP and their practice.[17] Data collection is evenly distributed throughout 50 weeks each year, allowing for two weeks' closure over the Christmas period. Only GPs who claimed at least 375 general practice Medicare items of service in the previous quarter are eligible to participate. Of the GPs who agree to participate each year, about 80% complete the project. The age–sex distribution of patients at encounters included in the BEACH study for whom a Medicare rebate was to be claimed has been repeatedly shown to accurately represent the age-sex distribution of patients at all GP service encounters for which Medicare claims have been made.[17] Problems managed at the encounter are secondarily coded by trained clinical coders using the International Classification of Primary Care (version 2) (ICPC-2) PLUS interface terminology[18] and automatically classified to ICPC-2.[19]

We used three approaches to test whether there had been a change in the management rate of genital warts, since the introduction of the vaccination program. First, we compared the rate of genital wart management in July 2002–June 2006 (Pre-program) with that in July 2008–June 2012 (Post-program). We excluded the year before and after the introduction date (1^st^ July 2007) to provide time for the vaccine to take effect (as genital wart incubation period is rarely longer than a year)[20]. The management rate was calculated using the surveymeans procedure is SAS 9.3 (SAS Institute Inc, Cary, NC, USA). We examined the management rate for each sex in three age groups (15–27 years, 28–49 years, 50 years and older). The age group 15–27 years at encounter was chosen because, by the end of June 2008, this age group would include all women of vaccine eligible age and hence potentially covered under the immunisation program. The patients aged 28–49 years were patients who may have had sexual contact with people covered by the program. Patients aged 50 years or over were considered unlikely to have sexual contact with patients covered in the program.

We then looked at the trend in management rate of genital warts by financial year in the pre-vaccine period (July 2000-June 2007) and in the post-vaccine period (July 2007-June 2012) using a linear regression analysis.

Lastly, the data were analysed from July 2000–June 2012 in two year periods. Two year data periods were used to increase the power of each observation as the frequency of genital warts management at encounters recorded in BEACH was relatively low.

Genital warts were defined as ICPC 2 codes Y76 for males and X91 for females. In all three analyses, we also examined: the management rate of genital herpes (ICPC-2 code X90) and gardnerella/bacterial vaginosis (ICPC-2 PLUS codes X84006, X84003) as control conditions for women; the management rate of genital herpes (ICPC-2 codes Y72) and urethritis (ICPC-2 codes U72) as control conditions for men. Chlamydia was not used as a control, as there had been a well-documented rise in its management over the study period – possibly due to increased testing for chlamydia.[21]

The BEACH study uses a single-stage cluster design, with a cluster of 100 patient encounters around each GP. In all analyses, we adjusted for this cluster using survey procedures in SAS 9.3 statistical software (SAS Institute Inc, Cary, NC, USA). The statistical significance of difference between two management rates was determined by non-overlapping 95% confidence intervals. This provides a more conservative estimate of significant difference than the 5% level, reducing the risk of Type I error, but increasing the risk of Type II error.

### Ethics Statement

During the data collection period for this study the BEACH program was approved by the Human Research Ethics Committee of the University of Sydney and the Ethics Committee of the Australian Institute of Health and Welfare. Our method involves the collection of data from unidentifiable, consenting patients. A patient information card is supplied in the research kit, which GPs are instructed to show to patients in order to obtain informed consent (an example shown in Britt et al[17]). If the patient chooses not to participate their encounter details are not recorded. GPs are asked not to provide written consent to the research body, as this prevents patients remaining anonymous. These methods comply with the Ethics requirements for the BEACH program.

## Results

Between July 2000 and June 2012, 11,780 GPs took part in the BEACH project, together recording 1,175,879 encounters with patients. On average, genital warts were managed at a rate of 0.9 per 1,000 encounters across this period (95% CI: 0.8–1.0). In the Pre-program period, patients aged 15–27 years had the highest rate of genital warts management (4.3 per 1,000 encounters for female and 4.9 for males), while patients aged 50 years and older had the lowest management rate (Table 1). This pattern of higher management rates in younger patients was also found for the control conditions. Male patients aged 28 years and older had a significantly higher management rate of genital warts than their female peers. We found no statistically significant difference between the management rate of genital warts at encounters with male and female patients aged 15–27 years.

**Table 1:** Management rate of genital wart and control conditions in general practice per 1,000 encounters Pre and Post quadravalent vaccine program introduction.

Patient age and sex groups	Genital warts per 1,000 encounters		Control conditions per 1,000 encounters	
n's = (Pre/Post)	Pre (July 2002- June 2006)	Post (July 2008-June 2012)	Pre (July 2002- June 2006)	Post (July 2008-June 2012)
**Female**				
15–27 years (n = 43,596/42,393)	4.33 (3.43–5.23)	1.67 (1.20–2.13)	5.04 (4.24–5.84)	6.73 (5.73–7.74)
28–49 years (n = 99,477/98,550)	1.25 (0.97–1.54)	0.80 (0.58–1.02)	4.02 (3.51–4.54)	4.75 (4.18–5.31)
50+ years (n = 186,015/205,046)	0.05 (0.01–0.09)	0.05 (0.01–0.09)	0.88 (0.68–1.07)	1.29 (1.07–1.51)
**Male**				
15–27 years (n = 21,157/18,745)	4.86 (3.74–5.98)	4.64 (3.47–5.81)	3.24 (2.36–4.12)	2.89 (2.01–3.77)
28–49 years (n = 55,711/52,120)	2.45 (1.91–2.98)	2.08 (1.59–2.57)	3.38 (2.71–4.04)	3.04 (2.41–3.67)
50+ years (n = 127,380/139,790)	0.27 (0.15–0.38)	0.21 (0.11–0.31)	0.77 (0.56–0.98)	0.85 (0.65–1.05)

Note: Control conditions for female patients were genital herpes (ICPC-2 code X90) and gardnerella/vaginosis (ICPC-2 PLUS codes X84006, X84003) and control conditions for male patients were genital herpes (ICPC-2 codes Y72) and urethritis (ICPC-2 codes U72).

The simple overall comparison in Table 1 shows that the only significant change in the management rate of genital warts between the Pre and Post program periods was a large 61% decrease in the management rate at encounters with female patients aged 15–27 years (Table 1). There was no change in the management rate of genital warts in older females or in males of any age group. For the control conditions, the only significant change between time periods was an increase in their management rate at encounters with women aged 50 years and over.

The trend analyses showed that after the introduction of the HPV vaccine, there was a significant year-on-year reduction in the management rate of genital warts in women aged 15–27 years, with the reduction being 71 fewer managements per 100,000 encounters per year (Table 2). During the same period, there was a significant increase in the management rate of control STIs for women in this age group, with an average increase of 97 managements per 100,000 encounters per year. There was a slight increase in the management rate of genital warts by year in male patients aged 50 years and over. There were no other significant trends in the post vaccine period.

**Table 2:** Trends in management rate of genital warts and control STIs in the Pre and Post vaccine periods by patient age and sex.

Age and sex group	Genital wart trend Pre-vaccine Effect of year per 100,000 encounters (p value)	Genital wart trend Post-vaccine Effect of year per 100,000 encounters (p value)	Control STI trend Pre-vaccine Effect of year per 100,000 encounters (p value)	Control STI trend post-vaccine Effect of year per 100,000 encounters (p value)
Females aged 15-27 years	28.95 (p = 0.064)	−71.47 (p <0.0001)*	36.69 (p = 0.0251)*	96.67 (p = 0.0027)*
Females aged 28-49 years	4.34 (p = 0.3695)	−1.00 (p = 0.8991)	31.91 (p = 0.0009)*	−2.16 (p = 0.9054)
Females aged 50+	0.70 (p = 0.5261)	−0.28 (p = 0.8631)	12.87 (p = 0.0002)*	9.73 (p = 0.1720)
Males aged 15–27 years	28.80 (p = 0.1160)	−20.96 (p = 0.5853)	1.48 (p = 0.9347)	7.04 (p = 0.7888)
Males aged 28–49 years	28.77 (p = 0.0080)*	2.67 (p = 0.8735)	12.16 (p = 0.2719)	−19.57 (p = 0.3229)
Males aged 50+	2.23 (p = 0.2170)	6.94 (p = 0.0082)*	0.20 (p = 0.9574)	−4.45 (p = 0.4646)

*Significant at the p < 0.05 level.

Note: Pre-vaccine period was July 2000-June 2007 and post vaccine period was July 2007-June 2012.

Male control STIs were genital herpes (ICPC-2 codes Y72) and urethritis (ICPC-2 codes U72).

Female control STIs were genital herpes (ICPC-2 code X90) and gardnerella/bacterial vaginosis (ICPC-2 PLUS codes X84006, X84003).

**Figure 1:**
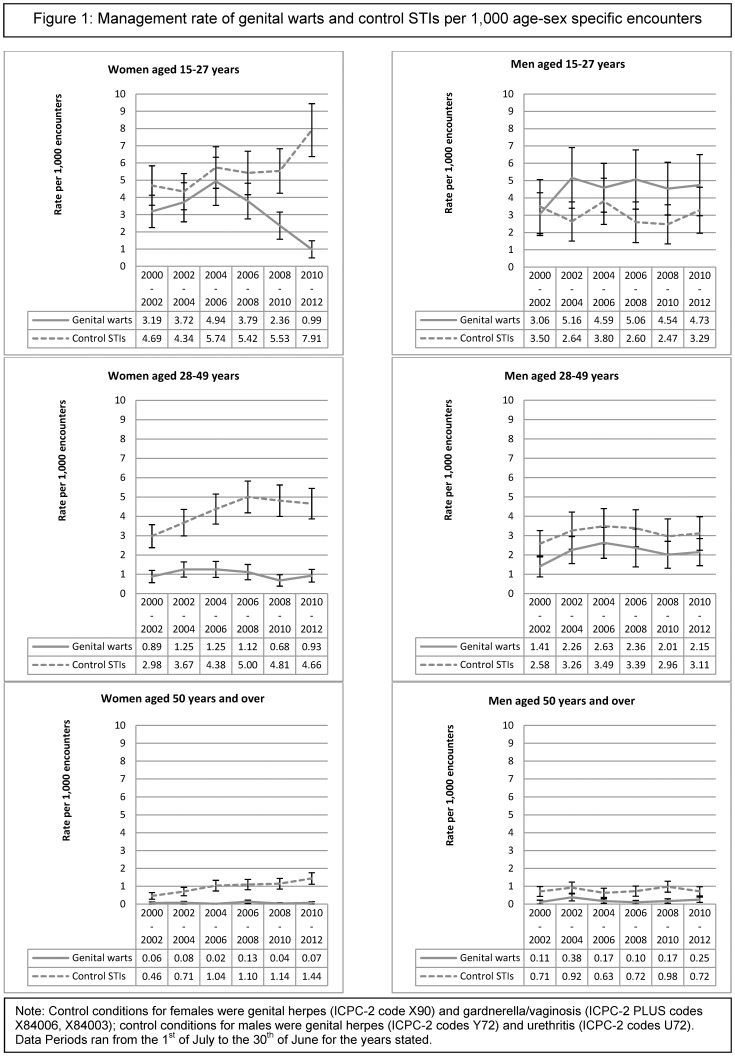
Management rate of genital warts and control STIs per 1,000 age-sex specific encounters.

Figure 1 graphically depicts the management rate of genital warts and the control conditions per 1,000 GP encounters between July 2000 and June 2012 in two year periods. For most age-sex groups, little changed over the study period in the management rates of both groups of problems. However, the significant decrease in the management rate of genital warts and the significant increase in control STI management for female patients aged 15–27 years are clearly demonstrated. It also shows a steady increase in the management rate of control conditions over the study period for women aged 50 years and older.

## Discussion

In our study we showed a significant decrease in the GP management rate of genital warts among women of vaccine-eligible age to be covered by the Australian national HPV vaccination program. These findings are the first from general practice which provides services to the majority of the Australia public, and supplement the findings from research in the small proportion of Australians who attend sexual health services. Our study indicates that the marked reductions in cases of genital warts in vaccine-eligible young women managed in sexual health services[15] are also reflected in vaccine eligible young women in the broader Australian population.

We did not find a reduction in management rates of genital warts in young men of the same age group as the vaccine-eligible young women. This is in contrast to the findings in sexual health clinics of a 36% reduction in diagnosis rates of genital warts in males aged less than 21 years and a 19% reduction in males aged 21–30 years.[15] One possible explanation for our lack of observed changes with men is that we did not collect information on the sex of the patient's partner, and so we were are not able to separate men who have sex with men in the analysis, which could have potentially influenced our results.

We saw a significant increase in management rates of gardnerella/vaginosis and/or genital herpes in women aged 50+ in the simple comparison. This suggests that the sexual risk of this group rose over time, although both genital herpes and bacterial vaginosis are conditions that relapse commonly and so presentations for these conditions do not necessarily indicate a change in sexual risk behaviour. The rate of genital warts in this group was extremely low and limited our statistical power to determine if rates changed substantially in this group. However other studies would suggest that the vaccination program would have been unlikely to have influenced the incidence of genital warts in this group.[13], [14]

We also saw a significant, though small, increasing trend in the management of genital warts in males aged 50+ after the introduction of the vaccination program. However, there was no significant difference found between the pre and post periods in the simple comparison. The increase in the trend analysis is probably due to there being no recorded management of genital warts in men of this age in BEACH between July 2007 and June 2008. As with women of the same age, we would not expect the vaccination program to have any effect on this group.

The strength of this study is that it is a nationally representative sample of general practice patient encounters from an ever changing sample of GPs. Even though management of genital warts in general practice is a relatively rare event, the majority (57%) of genital warts managed by a health professional are managed by a GP.[16] Only a study of the size of BEACH could have the power to test for any change in management in Australian general practice.

The study also has limitations. The patients' sexual history was not recorded, so we were unable to identify male patients who have sex with men (MSM). It is possible that if we could have identified the MSM patients, we may have found a similar decrease among the exclusively heterosexual male patients identified in the Donovan and Ali studies.[13], [14]

Further, by our 2012 data some women in the 28–49 year age group (those aged 28–32 years) would have been eligible for the catch-up program. We compared the two periods by patient age at the encounter, rather than calculated age at July 2007, so that we were comparing women of the same age and probable sexual experience. This means some women from the vaccination eligible group were included with non-vaccine eligible women, but as there was no significant change for the 28–49 year old group, it is unlikely that it had an effect on the outcome of the study.

## Conclusion

This study provides evidence that the quadrivalent HPV vaccination program has led to a decreased management rate of genital warts in general practice among those women of vaccine-eligible age. This study, along with those showing the decrease in diagnosis and management of genital warts in sexual health clinics and private hospitals suggests an overall community wide decrease in both the incidence of genital warts and its subsequent management. Due to this reduction, some young women in Australia have been spared the distress of having genital warts and the health system spared the cost of having to treat them. While this study did not find a significant decrease in genital warts among male patients attending general practice, it provides a baseline prior to the roll out of a similar scheme for boys. In future research, the BEACH program will allow us to assess whether the male focussed program has the same effect as the earlier female focussed program.

## References

[1] Walboomers JM, Jacobs MV, Manos MM, Bosch FX, Kummer JA, et al (1999) Human papillomavirus is a necessary cause of invasive cervical cancer worldwide. J Pathol 189: 12–19. 10451482 10.1002/(SICI)1096-9896(199909)189:1<12::AID-PATH431>3.0.CO;2-F

[2] Munoz N, Bosch FX, de SS, Herrero R, Castellsague X, Shah KV, et al (2003) Epidemiologic classification of human papillomavirus types associated with cervical cancer. N Engl J Med 348: 518–527. 12571259 10.1056/NEJMoa021641

[3] Lacey CJ, Lowndes CM, Shah KV (2006) Chapter 4: Burden and management of non-cancerous HPV-related conditions: HPV-6/11 disease. Vaccine 24 Suppl 3 S3–35-S3/41. 10.1016/j.vaccine.2006.06.015 16950016

[4] Aubin F, Pretet JL, Jacquard AC, Saunier M, Carcopino X, et al (2008) Human papillomavirus genotype distribution in external acuminata condylomata: a Large French National Study (EDiTH IV). Clin Infect Dis 47: 610–615. 18637758 10.1086/590560

[5] Garland SM, Steben M, Sings HL, James M, Lu S, et al (2009) Natural history of genital warts: analysis of the placebo arm of 2 randomized phase III trials of a quadrivalent human papillomavirus (types 6, 11, 16, and 18) vaccine. J Infect Dis 199: 805–814. 19199546 10.1086/597071

[6] Pirotta M, Ung L, Stein A, Conway EL, Mast TC, et al (2009) The psychosocial burden of human papillomavirus related disease and screening interventions. Sex Transm Infect 85: 508–513. 19703844 10.1136/sti.2009.037028

[7] Pirotta M, Stein AN, Conway EL, Harrison C, Britt H, Garland S (2010) Genital warts incidence and healthcare resource utilisation in Australia. Sex Transm Infect 86: 181–186. 19965802 10.1136/sti.2009.040188

[8] Brotherton JM, Deeks SL, Campbell-Lloyd S, Misrachi A, Passaris I, et al (2008) Interim estimates of human papillomavirus vaccination coverage in the school-based program in Australia. Commun Dis Intell Q Rep 32: 457–461. 19374275 10.33321/cdi.2008.32.45

[9] Leask J, Jackson C, Trevena L, McCaffery K, Brotherton J (2009) Implementation of the Australian HPV vaccination program for adult women: qualitative key informant interviews. Vaccine 27: 5505–5512. 19619501 10.1016/j.vaccine.2009.06.102

[10] Victorian Cytology Service Incorporated (2014 February) National HPV Vaccination Program Register. http://www.hpvregister.org.au/research/coverage-data/vaccination-2012. Accessed 2014 June 30^th^

[11] Agius PA, Pitts MK, Smith AM, Mitchell A (2010) Human papillomavirus and cervical cancer: Gardasil vaccination status and knowledge amongst a nationally representative sample of Australian secondary school students. Vaccine 28: 4416–4422. 20434543 10.1016/j.vaccine.2010.04.038

[12] The Hon Tanya Pilbersek MP Minister for Health Australia (2012 July) HPV vaccine extended to boys. Available: http://www.health.gov.au/internet/ministers/publishing.nsf/Content/mr-yr12-tp-tp059.htm. Accessed 2014 January 30^th^

[13] Donovan B, Franklin N, Guy R, Grulich AE, Regan DG, et al (2011) Quadrivalent human papillomavirus vaccination and trends in genital warts in Australia: analysis of national sentinel surveillance data. Lancet Infect Dis 11: 39–44. 21067976 10.1016/S1473-3099(10)70225-5

[14] Ali H, Donovan B, Wand H, Read TR, Regan DG, et al (2013) Genital warts in young Australians five years into national human papillomavirus vaccination programme: national surveillance data. BMJ 346: f2032. 23599298 10.1136/bmj.f2032

[15] Ali H, Guy RJ, Wand H, Read TR, Regan DG, et al (2013) Decline in in-patient treatments of genital warts among young Australians following the national HPV vaccination program. BMC Infect Dis 13: 140. 23506489 10.1186/1471-2334-13-140 PMC3606327

[16] Grulich AE, de Visser RO, Smith AM, Rissel CE, Richters J (2003) Sex in Australia: sexually transmissible infection and blood-borne virus history in a representative sample of adults. Aust N Z J Public Health 27: 234–241. 14696717 10.1111/j.1467-842x.2003.tb00814.x

[17] Britt H, Miller GC, Henderson J, Charles J, Valenti L, et al.. (2012) General practice activity in Australia 2011-12. Sydney: Sydney University Press.

[18] Britt H (1997) A new coding tool for computerised clinical systems in primary care–ICPC plus. Aust Fam Physician (Suppl 2): S79–S82. 9254947

[19] Classification Committee of the World Organization of Family Doctors (1998) ICPC-2: International Classification of Primary Care. Oxford: Oxford University Press.

[20] Oriel JD (1971) Natural history of genital warts. Br J Vener Dis 47: 1–13. 5550858 10.1136/sti.47.1.1 PMC1048137

[21] Sawleshwarkar S, Harrison C, Britt H, Mindel A (2010) Chlamydia testing in general practice in Australia. Sex Health 7: 484–490. 21062591 10.1071/SH09110

